# Chaos and confusion in *Hikikomori* research. Commentary on “The suitability of outing frequency as a definition of hikikomori (prolonged social withdrawal)”

**DOI:** 10.3389/fpsyt.2023.1199359

**Published:** 2023-05-17

**Authors:** Simone Amendola

**Affiliations:** Department of Applied Psychology, Zurich University of Applied Sciences, Zurich, Switzerland

## 1. Introduction

The recent article of Nonaka and Sakai (1) entitled “The suitability of outing frequency as a definition of hikikomori (prolonged social withdrawal),” published in Frontiers in Psychiatry (Section of Psychopathology) represents a significant analysis of the usefulness of outing frequency for the definition of hikikomori. Previously published data were used for a secondary analysis focused on the number of days individuals went out per week and qualitative indicators of outings as indicated by going to places that require or do not require interpersonal interactions. The definition of hikikomori (a condition characterized by a lack of social participation, which includes working, attending school, socializing outside one’s home, and staying at home on most days except for solitary outings for over 6 months) of the Ministry of Health, Labor, and Welfare was partially used. Of participants who reported being in a hikikomori condition, 28%–48% went out less than 1 day/week while 14%–21% went out 4 or more days/week. Correlation analyses found that, in hikikomori, outing frequency was positively associated with both going to places that require and do not require interpersonal interactions, whereas it was not associated with subjective social impairment. Furthermore, regression analyses indicated that low (less than 1 day/week) and medium (1–4 days/week) frequencies of outings compared with high (4 or more days/week) and subjective social impairment, decreased the probability of being in the control or recovered group compared with the hikikomori group. Conversely, going to places that require interpersonal interactions increased the probability of being in the control or recovered group compared with the hikikomori group. These results were consistent with the definition of hikikomori used, whereas going out freely and going to places that do not require interpersonal interactions showed no significant effect. Finally, the authors found that an outing frequency of 4.5/5 days/week was able to discriminate between the hikikomori and control groups.

### 1.1. Confusion in results interpretation

The authors discussed their results as initial evidence of the validity of the hikikomori criteria, which were proposed by Kato et al. (2, 3), misinterpreting their findings because a different hikikomori definition was used. The authors stated that “the cutoff points supported the criteria for the number of days outside the home proposed in previous studies” (p. 5). However, Kato et al. (2) defined the following required criteria for hikikomori: marked social isolation in one’s home; duration of continuous social isolation for at least 6 months; and significant functional impairment or distress associated with the social isolation. Furthermore, “individuals who leave their home frequently (4 or more days/week), by definition, do not meet criteria for hikikomori” (p. 431). Therefore, this definition is broader than the one used by Nonaka and Sakai, which also considered a lack of social participation (working, attending school), socializing outside one’s home, and reduced outings to solitary ones. Consequently, the cutoff of 4/5 days/week may not be valid when applied to a different hikikomori definition. For example, if applied to Kato et al.’s (2) definition may increase false positive cases.

Nonaka and Sakai noted that “the frequency distribution of outings showed that 14.5%–20.6% of those included by the previous definition would not be considered to have hikikomori” (p. 5). The authors refer to 14%–21% of hikikomori individuals who went out 4 or more days/week suggesting that those were false negative cases of hikikomori if the definition of Kato et al. (2, 3) was applied. Again, it needs to be considered that the two hikikomori definitions differ, and therefore, findings related to the use of one of these definitions may not apply to the other. A non-negligible proportion of hikikomori individuals (based on the single-item measure) went out more than 4 days/week. However, despite the question examining the presence of hikikomori mentioned “solitary outings,” questions exploring outing frequency and going to places did not. Therefore, it is not obvious that outing frequency measured only solitary outings. This limitation could have thus increased false positives according to the hikikomori definition by Nonaka and Sakai (“… except for solitary outings”).

In addition to the fact that the questions did not specifically explore solitary outings but rather outing frequency and going to places that overall require or do not require interpersonal interaction, one could argue what places that require or do not require interpersonal interaction refer to. To avoid subjective interpretations, future studies may provide explanations and examples for “places” and “interpersonal interaction.” Additionally, the use of variables (e.g., going out freely and going to places) with less than five categories as continuous scores may be questioned (and sensitivity analyses treating them as categorical/ordinal could have been valuable). The validity of the cutoff may be also challenged because both outing frequency and hikikomori were measured using single-item questions, and it is not clear whether the non-normal distribution of outing frequency was considered (4, 5). Notably, the study most suffered the absence of a valid measurement or gold standard for the detection of hikikomori (e.g., 25-item Hikikomori Questionnaire, clinical interview).

### 1.2. Chaos in hikikomori definition

Despite the above limitations, the findings of Nonaka and Sakai demonstrated the importance of lack of social interaction and social impairment as hikikomori characteristics. The authors suggested that “the lack of social interaction should be characterized as one of the hikikomori conditions” (p. 5). This is a point that deserves further attention. Two studies conducted in Hong Kong showed different frequencies of hikikomori, 1.9% (6) and 5% (7). In addition to different sampling designs, the diverse definitions of hikikomori used by the authors may explain the difference between the results of the two studies. Fong et al. (7) adopted the definition proposed by Kato et al. (2, 3) that does not include avoidance of social participation and interaction among the core criteria for hikikomori. In addition, the presence of other mental disorders does not constitute an exclusion criterion for the diagnosis of hikikomori. On the contrary, Wong et al. (6) partially adopted the definition of hikikomori of Teo and Gaw (8), including the following criteria: spending most of the day and nearly every day confined at home, persistent avoidance of social participation (such as going to school or working) and social relationships (such as friendships and contact with family members), exclusion of some mental disorders (i.e., social phobia, major depression, schizophrenia, and avoidant personality disorder), and duration of the social withdrawal behaviors of at least 6 months. Notably, the Cabinet Office of Japan (9) considers the following exclusion criteria for hikikomori: “Individuals whose current state had been triggered by an illness, such as schizophrenia or a physical disease; those who were pregnant or had recently given birth; those who worked from home; and those who were taking care of their children’s education […] those who stayed home but described themselves as a “housewife/husband” or “cleaner” […]” (p. 105). Future research needs to examine how the use of different criteria adequately represents the phenomenological presentation of hikikomori influencing its interpretation (10).

## 2. Discussion

The study by Nonaka and Sakai has the merit of representing an initial empirical test of the validity of outing frequency for the characterization of hikikomori, promoting additional research. When interpreting research findings, the potential impact of specific hikikomori definitions and contextual factors (11–13) should be examined. In accordance with the previous literature (8, 9, 14, 15), considering avoidance, disinterest, or unwillingness to attend school/work and participate in social relationships/interactions for the definition of hikikomori may help in going beyond a behavioral symptom (i.e., physical isolation), providing a useful psychological indicator of dysfunction (12) for hikikomori, besides impairment/distress and exclusion criteria described above. A recent systematic review (10) demonstrated a substantial agreement on the need to consider not working and attending school and poor socialization outside one’s home when studying hikikomori: more than 80% of the examined studies included not working or attending school, not socializing outside one’s home, and duration of hikikomori (generally, longer than 6 months) as indicators of hikikomori. The application of broad hikikomori definitions and lack of implementation of useful exclusion criteria may result in over-pathologization and over-diagnosis of individuals (e.g., those with a longstanding medical illness, disability, or functional impairment and houseworkers), showing social isolation for reasons other than persistent avoidance of or unwillingness to engage in social participation (such as going to school or working) and social relationships (such as friendships and contact with family members). Therefore, researchers need to consider the risk of confusing hikikomori with social isolation. Finally, after considering all the above evidence, the use of a valid measure of hikikomori symptoms such as the 25-item Hikikomori Questionnaire (16) may inform the study of the main characteristics of the condition, i.e., difficulty in socialization, preference for being alone, isolation, and poor emotional support from a dimensional perspective.

## Author contributions

The author confirms being the sole contributor of this work and has approved it for publication.

## Funding

SA is supported by a Swiss Government Excellence Post-Doctoral Scholarship [2022.0033] to conduct a meta-analysis of longitudinal studies on the associations between gaming disorder, internalizing symptoms of psychopathology and psychological distress. This funder had no role in study design, data collection, data analysis, data interpretation, or writing of the report. Open access funding by Zurich University of Applied Sciences (ZHAW).

## Conflict of interest

The author declares that the research was conducted in the absence of any commercial or financial relationships that could be construed as a potential conflict of interest.

## Publisher’s note

All claims expressed in this article are solely those of the authors and do not necessarily represent those of their affiliated organizations, or those of the publisher, the editors and the reviewers. Any product that may be evaluated in this article, or claim that may be made by its manufacturer, is not guaranteed or endorsed by the publisher.
